# The C-terminus hot spot region helps in the fibril formation of bacteriophage-associated hyaluronate lyase (HylP2)

**DOI:** 10.1038/srep14429

**Published:** 2015-09-23

**Authors:** Harish Shukla, Sudhir Kumar Singh, Amit Kumar Singh, Kalyan Mitra, Md. Sohail Akhtar

**Affiliations:** 1 Molecular and Structural Biology Division, CSIR-Central Drug Research Institute, Sector 10, Jankipuram Extension, Lucknow, INDIA, PIN 226 03; 2 Sophisticated Analytical Instrument Facility , CSIR-Central Drug Research Institute, Sector 10, Jankipuram Extension, Lucknow, INDIA, PIN 226 031; 3 Academy of Scientific and Innovative Research, CSIR-Central Drug Research Institute, Sector 10, Jankipuram Extension, Lucknow, INDIA, PIN 226 031

## Abstract

The bacteriophage encoded hyaluronate lyases (HylP and HylP2) degrade hyaluronan and other glycosaminoglycans. HylP2 forms a functional fibril under acidic conditions in which its N-terminus is proposed to form the fibrillar core, leading to nucleation and acceleration of fibril formation. Here we report the presence of a hot spot region (A_144_GVVVY_149_) towards the carboxy terminus of HylP2, essential for the acceleration of fibril formation. The ‘hot spot’ is observed to be inherently mutated for valines (A_178_AMVMY_183_) in case of HylP. The N- terminal swapped chimeras between these phage HLs (^N^HylP_2_^C^HylP and ^N^HylP^C^HylP2) or HylP did not form fibrils at acidic pH. However, seeding of prefibrils of HylP2 recompensed nucleation and led to fibrillation in ^N^HylP^C^HylP2. The V147A mutation in the ‘hot spot’ region abolished fibril formation in HylP2. The M179V and M181V double mutations in the ‘hot spot’ region of HylP led to fibrillation with the seeding of prefibrils. It appears that fibrillation in HylP2 even though is initiated by the N-terminus, is accelerated by the conserved ‘hot spot’ region in the C-terminus. A collagenous (Gly-X-Y)_10_ motif in the N-terminus and a mutated ‘hot spot’ region in the C-terminus of HylP affect fibrillar nucleation and acceleration respectively.

Protein aggregates are generally derived from certain structural changes within a soluble protein or peptide. These aggregation prone β-sheet enriched species are crucial in the formation of fibrils or amyloid plaques and are characteristics of neurodegenerative disorders such as Alzheimer and Parkinson disease^1^,^2^,^3^,^4^,^5^. It is widely recognized that fibril formation in globular proteins generally proceeds through an “amyloidogenic intermediate” arising as a result of mutations or changes in the solution conditions^6^,^7^,^8^. The unfolded conformational state of a protein can easily enable the specific intermolecular interactions necessary for amyloid aggregation^6^,^9^,^10^. The amyloidogenicity of a protein has also been attributed to the presence of short amino acid segments from amyloid forming proteins, such as the six-residue segment of Tau protein and the seven-residue segment from yeast prion Sup35 protein^2^,^5^. Similarly, the insertion of a six residue fragment from the amyloidogenic SH3 domain of bovine phosphatidyl inositol 39 kinase to the non-amyloidogenic SH3 domain of a-spectrin was found to cause amyloid like fibril formation^11^. In addition, swapping of the aggregation prone segment also initiate aggregation, as observed in the case of human b2 microglobulin where transplantation of a seven residue segment to its homologous but non-amyloidogenic mouse b2 microglobulin created an amyloidogenic protein^3^. The mature fibrils generally lose their activity, though fibrillar structures in certain proteins fold as a functionally active fibril like in the case of nuclear protein controlling polyadenylation (PAPBN1), RNase A, phage hyaluronate lyase (HylP2) etc.^12^,^13^,^14^. These findings raise questions pertaining to fibrillar structure as to whether native-like structural domains undergo conformational modification or do they simply refold on fibril formation? Furthermore, understanding the mechanism of self-assembly of proteins into fibrils has been crucial in finding inhibitors of amyloid formation^15^,^16^.

The *Streptococcus pyogenes* bacteriophage encodes hyaluronate lyases (HLs) termed as HylP, HylP1 and HylP2^14^,^17^,^18^,^19^,^20^,^21^. These bacteriophage HLs, despite showing a high degree of similarity to each other (homology-82%, identity-62%), differ with respect to the presence of a collagen-like motif, (Gly-X-Y)_10_ present at the N-terminus of HylP^14^,^17^. The structure of phage HLs mainly consists of an N-terminal globular domain, followed by a triple-stranded β helix (TSβH) domain and a stretch of coiled coils with segmented α-helical nose at the C-terminus^19^,^20^. HylP2 forms functional fibrils at pH 5 through solvent exposed nonpolar surfaces and by intermolecular β-sheet formations. The partially unfolded N-termini of HylP2 associate together to form high molecular weight aggregates which later collapse into an ordered array that appears as a thin film. The proteolysed carboxy- terminus of HylP2 does not lead to fibrillation whereas the complex consisting of proteolytic fragments of N- and C-terminal domains form fibrils similar to the full length enzyme. It was therefore proposed that the N-terminus of HylP2 is essential for the nucleation as well as acceleration of fibril formation. The formation of functional fibril at pH 5, which is close to the optimum pH for enzyme activity is proposed to be advantageous during phage infection^14^.

Here we elucidate the details of fibril formation by carrying out the N-terminal domain swapping between HylP2 and HylP, together with the identification of a ‘hot spot’ region towards the C-terminus (in TSβH domain) responsible for the elongation of fibrils in HylP2.

## Results

### Purification of HLs, chimeras and mutants

**Figure 1:**
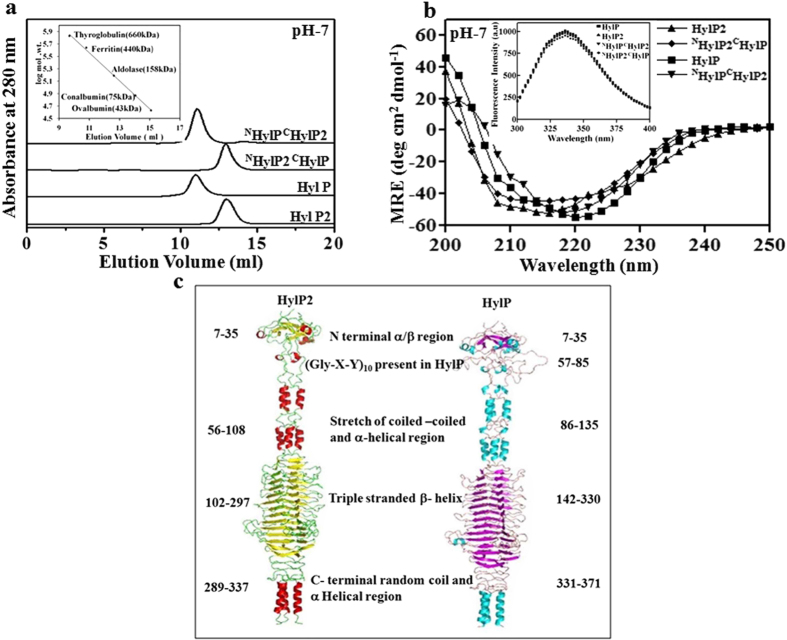
Structural changes in the chimeras. (**a**) The SEC profile of phage HLs and chimeras on Superdex 200 HR column at 25 °C. The inset shows the standard calibration curve run on similar condition. (**b**) The CD ellipticity at 218 nm for phage HLs and chimeras. The inset shows their relative tryptophan fluorescence intensity spectra with excitation at 285 nm. (**c**) The modelled structure of HylP2 and HylP using the structure coordinates of HylP2.

We aimed to comprehend the mechanism of fibril formation in HylP2 and swapped its N-terminal domain with the homologous HylP and vice versa (^N^HylP2^C^HylP and ^N^HylP^C^HylP2). The proteins were purified as described in the “methods”. The oligomeric assembly of the purified proteins was determined by size exclusion chromatography (SEC) at pH 7. The observed elution volumes of HylP2, HylP, ^N^HylP2^C^HylP and ^N^HylP^C^HylP2 were ~13.1 ml, ~11.1 ml, ~13.1 ml and ~11.1 ml respectively (Fig. 1a). The secondary and tertiary structures of the chimeras were determined by measuring the far UV circular dichroism (CD) spectrum and relative tryptophan fluorescence intensity (Fig. 1b and inset). It appeared that there were no significant changes in the corresponding structures between chimeras and native phage HLs. The modelled structures of HylP2 and HylP using the structure coordinates of HylP2^20^ are shown in Fig. 1c.

### Fibrillation in HylP2 is not accomplished with the N-terminal domain

**Figure 2:**
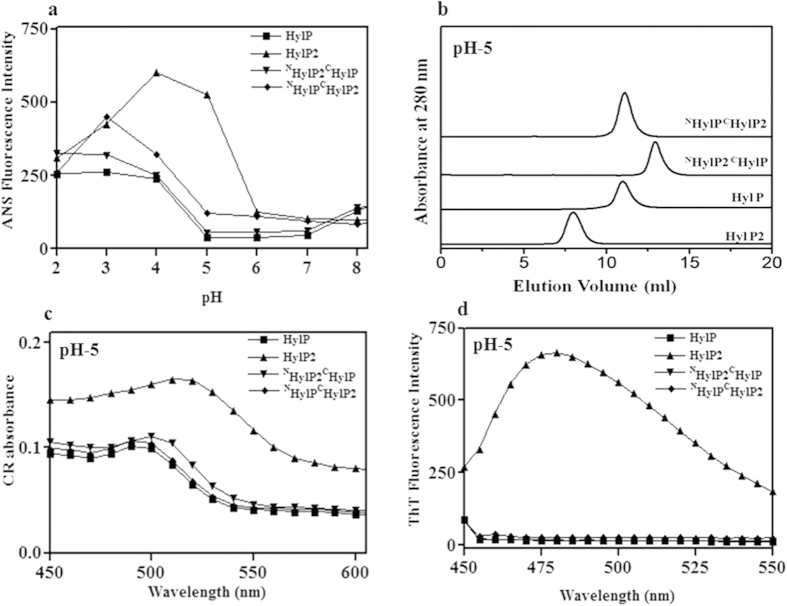
The N-terminus of HylP2 does not lead to fibril formation. (**a**) pH-dependent changes in the ANS fluorescence intensity in binding to phage HLs and their N-terminal swapped chimeras. (**b**) The SEC profile of phage HLs and chimeras on Superdex 200 HR column at 25 °C and pH 5. (**c**) Binding of CR to HLs or chimeras shows a red shift in the absorption maxima of the dye for HylP2 only (**d**) Binding of ThT to HLs or chimeras shows an enhancement in the fluorescence intensity for HylP2 only.

To study the partially unfolded species with solvent-exposed hydrophobic clusters, we performed 1-anilino-8-N-naphthalenesulfonic acid (ANS) binding affinity at 465 nm as a function of pH for HylP2, HylP, ^N^HylP2^C^HylP and ^N^HylP^C^HylP2 (Fig. 2a). The observed enhancement in ANS fluorescence intensity for HylP2 suggests a prominent exposure of its hydrophobic residues at lower pH range. HylP or the chimeras did not show significant ANS binding at pH 5.

The changes in the quaternary structure of above proteins were monitored at a condition where the HylP2 was stabilized in a partially unfolded condition and formed fibrils^14^. The SEC profiles of HylP2, HylP, ^N^HylP2^C^HylP and ^N^HylP^C^HylP2 at pH 5 show the elution volumes of ~8.2 ml, ~11.1 ml, ~13.1 ml and ~11.1 ml respectively (Fig. 2b). As is evident, there was no apparent change in the elution volume of HylP, ^N^HylP2^C^HylP and ^N^HylP^C^HylP2 between pH 5 or pH 7, while HylP_2_ showed a decrease in the elution volume from ~11.1 ml at pH 7 to ~8.2 ml at pH 5.

The existence of any fibrous texture in HylP, ^N^HylP2^C^HylP and ^N^HylP2^C^HylP were examined by monitoring their binding to dyes known to detect fibrils^22^. Congo red (CR) is a diazo dye that binds preferentially to the ordered, aggregated form of peptides/proteins with a red shift in its absorbance spectrum from 490 to 540 nm^23^,^24^. Thioflavin T (ThT) is a fluorescent dye that binds to the linear array of β-strands in amyloid fibrils with an enhancement in the fluorescence emission intensity^25^,^26^. Free ThT has excitation and emission maxima at 350 and 450 nm respectively. However, upon binding to fibrils the excitation and emission λ max change to 450 and 480 nm respectively.

Figure 2c shows the change in the absorption maxima with respect to the binding of CR at pH 5 to HylP2, HylP, ^N^HylP2^C^HylP and ^N^HylP2^C^HylP respectively. HylP2 showed a red shift of about 20–25 nm in the absorption maxima, while HylP, ^N^HylP2^C^HylP and ^N^HylP2^C^HylP did not. Figure 2d shows the changes in the emission intensity of ThT upon binding to HylP2, HylP, ^N^HylP2^C^HylP and ^N^HylP2^C^HylP at pH 5. The emission intensity for HylP, ^N^HylP2^C^HylP, ^N^HylP2^C^HylP remained unchanged and only HylP2 showed a significant enhancement in the fluorescence intensity at 480 nm.

### HylP2 contains an aggregation prone ‘hot spot’ region towards the C-terminus region

**Figure 3:**
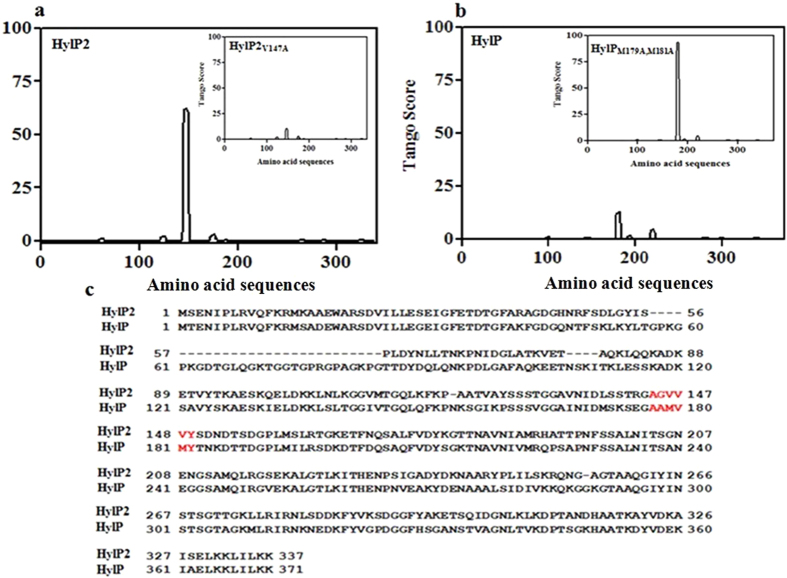
The C-terminus of HylP2 contains a hot spot region for fibrillation. (**a**) A hot spot region (**A**_**143**_**GVVVY**_**149**_) towards the C-terminus of HylP2 showing high Tango score (>60) of fibrillation. The inset shows that the V147A mutation in HylP2 leads to the abolishment of a high Tango score of fibrillation. (**b**) A non conserved hot spot region (**A**_**176**_**AMVMY**_**182**_) towards the C-terminus of HylP having an insignificant Tango score of fibrillation. The inset shows that the mutation of methionines to valines (**A**_**176**_**AVVVY**_**182**_) in HylP leads to the establishment of a high Tango score of fibrillation. (**c**) The sequence alignment of HylP2 and HylP showing the hot spot region (red) in HylP2 and HylP.

Some specific regions in proteins are known to act as ‘hot spots’ driving aggregation. This region is more relevant for proteins lacking significant secondary and tertiary structures or specific intra-chain interactions that could mask these aggregation-prone regions^27^. The role of individual residues in fibril formation and prediction of sensitive areas were investigated by a set of in silico experiments using algorithms that consider physicochemical properties of the proteins^28^,^29^,^30^. We examined the HylP2 sequence for ‘hot spot’ regions, using Tango software which predicted an aggregation prone region (A_144_GVVV_148_) with a high score of >60 (Fig. 3a). When *in silico* mutations were introduced in the ‘hot spot’ region, it was observed that V147A mutation leads to a drastic reduction in the Tango score from more than 60 to about 5 (Fig. 3a inset). The hot spot region in HylP is inherently mutated (A_176_AMVMY_182_) and has a very low Tango score for fibrillation. The *in silico* mutation of methionines to valines in the ‘hot spot’ region (A_176_AVVVY_182_) established the high Tango score of fibrillation (Fig. 3b and inset). Figure 3c shows the sequence alignments of HylP2 and HylP and the respective hot spot regions.

**Figure 4:**
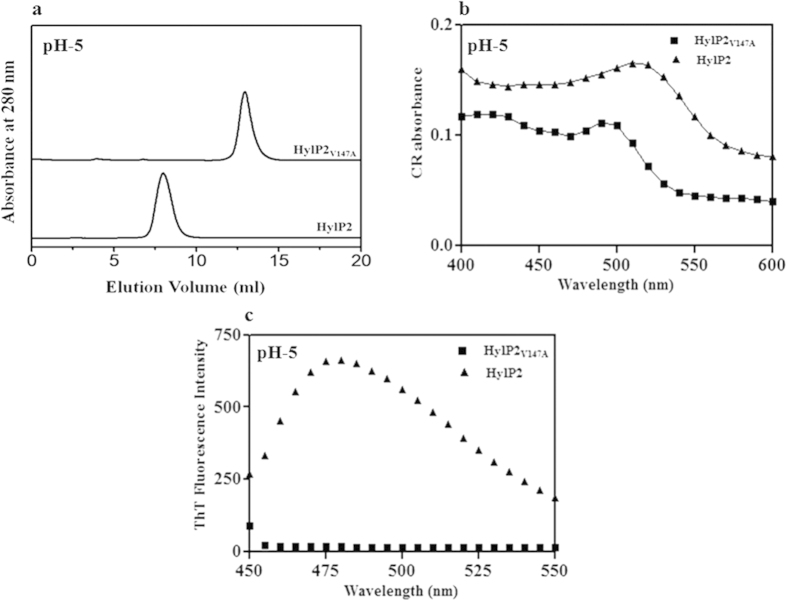
The V147A mutation in the hot spot region of HylP2 prevents fibrillation. (**a**) The SEC profile of HylP2_V147A_ on Superdex 200 HR column at 25 °C shows the absence of aggregation at pH 5. HylP2 on similar condition eluted in the void volume. (**b**) Binding of CR to HylP2 and HylP2_V147A_. Unlike for HylP2, CR does not interact with HylP2_V147A_ and there was no fibrillation. (**c**) Binding of ThT to HylP2 and HylP2_V147A_. Unlike for HylP2, ThT does not interact with HylP2_V147A_ and there was no fibrillation.

To test the effect of valine mutation in the aggregation of HylP2, we also created the desired point mutation (HylP2_V147A_) in the ‘hot spot’ region. The mutation did not affect the secondary, tertiary or oligomeric conformation of HylP2 at pH 7 (data not shown). The change in the oligomeric conformation and the formation of fibrils was subsequently analyzed at pH 5 (Fig. 4). In contrast to HylP2 which forms aggregates at pH 5, HylP2_V147A_ did not aggregate and the elution volume of HylP2_V147A_ (~11.1 ml) remained unchanged at pH 5 and pH 7 (Fig. 4a). Similarly, in contrast to HylP2 which showed strong binding to CR and ThT at pH 5, HylP2_V147A_ did not show significant interaction with these dyes (Fig. 4b,c). The result suggests a significant role of the ‘hot spot’ region in the fibril formation of HylP2.

### The N-terminal is involved in the initiation whereas the ‘hot spot’ region in the propagation of fibrillation in HylP2

**Figure 5:**
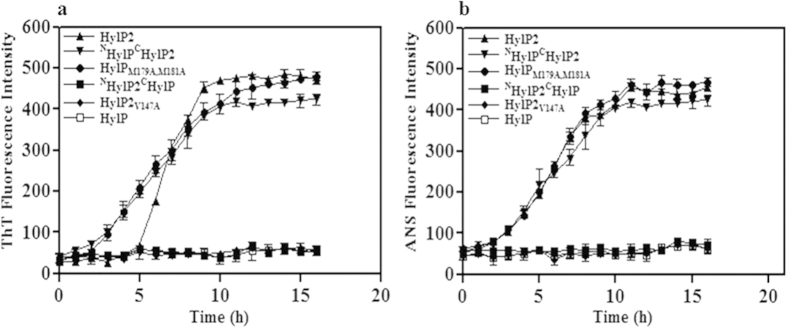
The fibrillation initiates from the N-terminus, but propagate due to the conserved hot spot region. (**a**) Kinetics of fibrillation pathway for phage HLs and its variants upon treatment with preformed fibrils and monitored by changes in the ThT fluorescence intensity as a function of time. ^N^HylP^C^HylP2 and HylP_M179V,M181V_ (HylP having methionine mutations in the hot spot region) formed fibrils upon seeding with preformed fibrils. HylP, ^N^HylP_2_^C^HylP and HylP2_V147A_ did not form fibrils at similar conditions. The error bars indicate the means ± S.E. (**b**) Changes in the ANS fluorescence intensity at 465 nm with respect to time upon seeding with preformed fibrils for HLs and its variants as shown above. The error bars indicate the means ± S.E.

To understand the fibril formation, we carried out time-dependent binding studies with ThT. The changes in the ThT fluorescence emission as a function of time was monitored to observe the rate of fibril formation for HylP2, HylP, ^N^HylP2^C^HylP, ^N^HylP^C^HylP_2_, HylP2_V147A_ (the hot spot mutant with abolished Tango score of fibrillation) and HylP_M179V,M181V_ (the hot spot mutant with re-established Tango score of fibrillation) (Fig. 5a). As reported, the increase in ThT fluorescence intensity for HylP2 fibrillation followed a sigmoidal pattern in which the nucleation phase (4 h) was followed by a log phase of elongation (6 h) and a plateau region of maturation^14^. The kinetics of fibril formation by ^N^HylP^C^HylP2 (lacking the N-terminal domain desired for nucleation, but having the C-terminal domain with the hot spot region desired for the acceleration) was initiated by adding preformed HylP2 fibrils. The seeding initiated fibrillation similar to that observed for HylP2. However, the chimera lacked the lag phase due to the presence of preformed fibrils. HylP_M179V,M181V_ also formed fibrils upon seeding of the prefibrils. This suggests that the elongation of the fibrils is facilitated by the ‘hot spot’ region. Under similar experimental conditions, ThT emission was not observed for HylP, ^N^HylP2^C^HylP and HylP2_V147A_ (Fig. 5a). The absence of fibrillar formation in these proteins was either due to the presence of collagenous Gly-X-Y motif at the N-terminus (for HylP) or the lack of the conserved ‘hot spot’ region at the C-terminus (for HylP/ ^N^HylP2^C^HylP/HylP2_V147A_), affecting the nucleation and/or elongation of fibril formation. The lack of fibril formation in the recombinant HylP2_127–337_ (data not shown) suggests that the fibrillation in phage HL is mediated by the N-terminus region where the high molecular weight aggregates collapse further into ordered fibrils^14^. Similar results of fibril formation with seeding of pre fibrils were obtained when the fibrillation was monitored using time dependent kinetic analysis for ANS binding (Fig. 5b).

**Figure 6:**
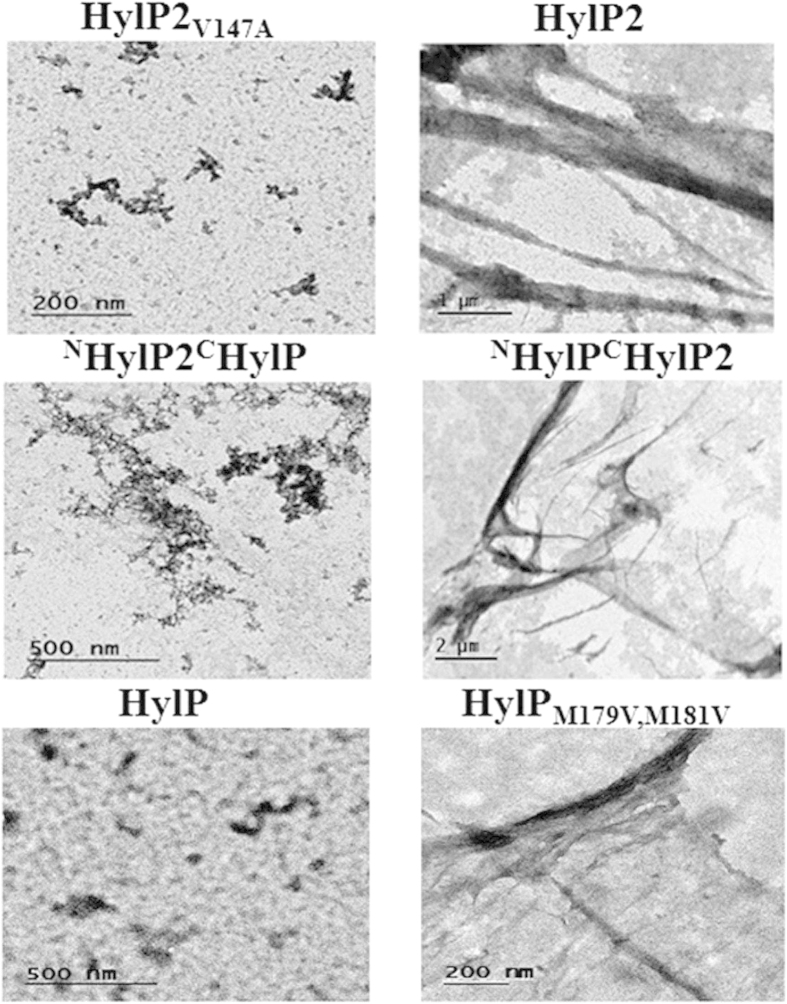
Transmission electron microscopy picture reveals thin fibrillar film like structures. HylP2_V147A_, ^N^HylP_2_^C^HylP and HylP did not form fibrils even after seeding with the preformed fibrils (left panel) where as ^N^HylP^C^HylP_2_ and HylP_M179V,M181V_ formed sheet-like structures (right panel).

Subsequently, fibril formation was studied using negative staining transmission electron microscopy (TEM) (Fig. 6). The HLs having (Gly-X-Y)_10_ motif at the N-terminus and/or the non conserved ‘hot spot’ region (HylP/ ^N^HylP_2_^C^HylP) or mutation which abolishes the high Tango score of fibrillation (HylP2_V147A_), did not form fibrils, even after seeding with preformed fibrils (Fig. 6. left panel). However, the HL variants with high Tango score of fibrillation (HylP_M179V,M181V_/ ^N^HylP^C^HylP_2_) formed fibrils and fibrillar bundles which further formed sheet-like structures with the seeding of preformed HylP2 fibrils (Fig. 6 right panel). This suggests that fibrillation can be achieved by bypassing the need for nucleation with the addition of preformed fibrils and their further elongation in presence of a ‘hot spot’ region with high Tango score for fibrillation. The sheet like structures observed for HylP_M179V,M181V_ and ^N^HylP^C^HylP_2_ are similar to the one observed for HylP2^14^.

## Discussion

The formation of protein fibrils by hyaluronidases was first reported for HylP2^14^. The enzyme formed a thin membrane like fibril resembling the structure formed by reflectin and amelogenin^14^,^31^,^32^. The N-terminal domain of HylP2 modulates the kinetics of fibrillation and is proposed to be essential for the formation as well as acceleration of fibrillation^14^. Here we examined the exclusive role of the N-terminal domain of HylP2 in fibrillation, in conjunction with the homologous HylP and their N-terminal swapped chimeras (^N^HylP_2_^C^HylP and ^N^HylP^C^HylP_2_). Similarity in the elution volumes of ^N^HylP_2_^C^HylP and ^N^HylP^C^HylP_2_ to HylP2 and HylP respectively suggests that the globular nature of the N-terminus of phage HLs may determine the quaternary structure. HylP2 was observed to bind to dyes CR and ThT that are used for the detection of amyloid aggregation, while HylP or the chimeras did not. These observations suggest that the N- terminus or the C-terminus of phage HLs does not lead to the fibril formation independently. The collagenous (Gly-X-Y)_10_ motif at the N-terminus of HylP is further known to affect its unfolding and helps in the stabilization^19^. We subsequently analyzed the region towards the C-terminus of HylP2 for the fibrillation properties and observed a ‘hot spot’ sequence (A_144_GVVVY_149_) having high Tango score of fibrillation. The sequence alignment with HylP shows that the corresponding region is mutated for valine (A_178_AMVMY_183_) resulting in a very low Tango score of fibrillation. The absence of aggregation for ^N^HylP_2_^C^HylP could thus be due to the mutation in the ‘hot spot’ region for HylP. The valine in the ‘hotspot’ region seems to be essential for retaining the high score of fibrillation and its mutation (HylP2_V147A_) abolished the aggregation properties of HylP2. In cases (^N^HylP^C^HylP2 or HylP_M179V_, _M181V_) where the nucleation is bypassed by seeding with preformed fibrils, the fibrillation process lack the lag phase and the fibril elongation is facilitated by the conserved ‘hot spot’ region. The lack of conserved ‘hot spot’ region for ^N^HylP2^C^HylP or HylP2_V147A_ affects the fibrillation.

In conclusion, this study suggests that the fibril formation in HylP2 is initiated at the N-terminus region and can accelerate only in presence of the conserved ‘hot spot’ region towards the C-terminus.

## Methods

### Cloning, site directed mutagenesis and preparation of proteins

The cloning and preparation of full length HylP_1–371_ and HylP2_1–337_ has been described previously^19^,^21^. The chimeras, where the N-terminus between HylP and HylP2 were swapped (^N^HylP_1–154_^C^HylP2_127–337_/ ^N^HylP^C^HylP2 and ^N^HylP2_1–126_^C^HylP_160–371_/^N^HylP_2_^C^HylP) were generated using primer pairs 5′CCCGCTAGCATGACTGAAAATATACCATTA3′, 5′ATTAAACCATCCTCGTCAAC GGGTGGAGCGGTCAATATT3′,5′CGACAAGTCAATATTGACCGCTCCACCCGTTGACGAGGA3′,5′CCCCTCGAGTTTTTTTAGTATGAGTTTTTT3/5′CCCGCTAGCATGAGTGAAAATATACCGCTG3′, 5′AAGCCAGCCGCCACTGTTGCTTATTCATCTTCCGTA GGA3′,5′AATCGCTCCTCCTACGGAAGATGAATAAGCAACAGTGGC3′, 5′CCCCTC GAGTTTTTTTAGTATGAGTTTTTT3′. HylP2_127–337_ was generated using primer pairs 5′CCGGCTAGCTCCTCGTC AACGGGTGGAGCGGTCAAT3′/ 5′CCCC TCGAGTTTTTTTAG TAT GAGTTTTTT3′. The amplification condition used were : 94 °C for 5 min; 94 °C for 30 s, 55 °C for 1 min, 68 °C for 3 min (30 cycles); 68 °C for 10 min. These amplified gene fragments were digested with NheI and XhoI and then ligated into the pET-23a (+) vector (Novagen) cut with the same enzymes. Competent *Escherichia coli* DH5-α cells were transformed with the plasmid constructs and screened for positive clones. The mutants (HylP2_V147A_ and HylP_M179V,M181V_) were generated from respective vector constructs using the GeneTailor™ Site-Directed Mutagenesis System (Invitrogen) with the mutagenic primer pairs 5′TCGGAAGGTGCTGCTGCTGTGGTGGTGTATACAAATAAAGAT3′, 5′ATCTT TATTTGTATACACCACCACAGCAGCACCTTCCGA3′/5′ACTAGAGGTGCTGGTGCT GGTGTTGCTGTCTATTCTGACAATGAT3′,5′ATCATTGTCAGAATAGACAGCAACACCAGCACCTCTA GT3). The conditions used for amplification were same as specified for use with Platinum *Pfx* DNA polymerase (Invitrogen). The DNA sequencing of all the amplified genes confirmed the homogeneity of the sequences. Subsequently the *E. coli* BL21 (DE3) were transformed with the resulting constructs for checking the expression. The condition used for the over expression and purification of chimeras and mutants was similar to, as described for HylP and HylP2. The homogeneity of the purified proteins was checked by SEC on a Superdex 200 HR 10/300 column (manufacturer’s exclusion limit 600 kDa) with AKTA fast performance liquid chromatography (Amersham Biosciences). The column was equilibrated with respective buffers at the desired pH before running the test samples. 500 μl of the sample was loaded on the column and run at 25 °C at a flow rate of 0.3 ml/min, and eluted protein was detected at 280 nm.

### Fibril formation

Phage HLs and its variants at a concentration of 1 mg/ml was dialyzed in 10 mM CGH buffer at pH 5. The dialysis was carried out at 4 °C for 24 h. Aliquots were taken at desired time intervals for monitoring the kinetics of fibril formation.

### CD measurements

CD measurements were made in a Jasco J-810 spectropolarimeter. The results were expressed as the mean residual ellipticity. Each spectrum was an average of three scans. Spectra were recorded using 3 μM of HLs and its variants in 1-mm cell, with 10 mM CGH buffer containing 150 mM sodium sulfate and 10% glycerol.

### Fluorescence measurements

Fluorescence spectra were recorded using a LS 50B spectrofluorometer (PerkinElmer) in a 5-mm path length quartz cell at 25 °C. For tryptophan fluorescence, excitation wavelength of 290 nm was used, and the spectra were recorded between 300 and 400 nm using 3 μM of HLs and its variants. For ANS binding studies, excitation wavelength was 350 nm, and the emission spectra were recorded between 400 and 500 nm. The final concentration of ANS used for the experiments was 10 μM. 5 μM aliquots of the dialyzing samples were added to a solution containing ANS in the dialyzing buffer at desired pH and mixed for 2 min before measuring the fluorescence emission. Background absorption of the buffer for the native proteins was subtracted from each reading. All readings were taken in triplicate.

### CR binding

The CR solutions were added to 5 μM of protein solutions (dialyzed at pH 5) to a final dye concentration of 10 μM and the samples were incubated for 2 min. The absorption spectrum of each sample was recorded from 400 to 700 nm on a UV-visible spectrophotometer using 1-cm path length quartz cuvette and corrected for contributions of buffer and protein. The spectrum of CR alone was compared with that of CR solutions in the presence of protein. Red shift together with an increase in absorption was taken to be indicative of the formation of fibrillar structures. All readings were taken in triplicate and the SD was calculated accordingly.

### ThT binding assay and kinetics of fibrillation

An aliquot of the HLs and its variants (5 μM) at pH 5 were added to a solution containing 10 μM ThT, and shaken for 5 min before measuring the fluorescence emission at 25 °C. A background fluorescence spectrum obtained by running a blank buffer with ThT was subtracted from each sample fluorescence spectrum. The excitation wavelength was 450 nm, and the emission was recorded at 480 nm. Fluorescence intensity at 480 nm was plotted against time for analysis. All readings were taken in triplicate.

### TEM studies

7 μl samples were deposited on freshly glow discharged carbon coated copper grids and allowed to adsorb. Excess solution was blotted off using a filter paper and negatively stained with 1% uranyl acetate (pH 4.2). The grids were air dried and observed under the TEM (FEI Tecnai Twin) at 80 kV after complete gun alignment and astigmatism correction. The images were acquired using a MegaView II CCD camera.

## Additional Information

**How to cite this article**: Shukla, H. *et al.* The C-terminus hot spot region helps in the fibril formation of bacteriophage-associated hyaluronate lyase (HylP2). *Sci. Rep.* **5**, 14429; doi: 10.1038/srep14429 (2015).
